# Study on static and dynamic mechanical properties and microstructure of silica fume-polypropylene fiber modified rubber concrete

**DOI:** 10.1038/s41598-024-63341-z

**Published:** 2024-05-31

**Authors:** Chenyue Han, Jianyong Pang, Shi Hu, Chunchun Yang

**Affiliations:** 1 School of Civil Engineering and Architecture, Anhui University of Science and Technology, Huainan, 232001 PR China; 2 Department of Structural Engineering, College of Civil Engineering, Tongji University, Shanghai, 200092 PR China; 3 Shanxi Research Institute for Clean Energy, Tsinghua University, Taiyuan, 030032 PR China

**Keywords:** Silica fume, Polypropylene fiber, Rubber concrete, Dynamic increase factor, Microstructure, Civil engineering, Composites

## Abstract

Through tests and micro-observations, the static and dynamic mechanical properties and microstructure of rubber concrete samples modified with varying amounts of silica fume and polypropylene fiber content were explored. The results indicate that incorporation of silica fume and polypropylene fiber can effectively enhance the performance of rubber concrete. Moreover, at 10% and 0.1% of silica fume and polypropylene fiber content respectively, rubber concrete’s compressive strength, splitting tensile strength, flexural strength, and dynamic compressive strength reached maxima. Furthermore, microstructure characteristic analysis indicated that inadequate adhesion between rubber particles and the matrix is responsible for compromised bearing capacity in unmodified rubber concrete. However, with the addition of silica fume and polypropylene fiber, the fiber binds the rubber particles closely with the matrix, while the silica fume fills the gaps between the matrix components. This combination results in rubber concrete with a denser internal structure and enhances its bearing capacity significantly.

## Introduction

Concrete is a critical pillar of civil construction, transportation, and water conservancy projects; however, concrete relies heavily on gravel and sand aggregates typically obtained through environmentally destructive methods including excavations of mountains and dredging of riverbeds^1^. Mass extraction of these aggregates not only depletes natural resources but also degrades affected parts of the environment^2^. As a consequence, finding sustainable alternatives to sand and stone aggregates is crucial to the long-term sustainable development of the concrete industry. In a similar but parallel vein, finding environmentally safe methods to handle “black pollution” or waste rubber from end-of-use tires is also a global concern, needing urgent and effective solutions^3^. One potential solution proposed is the use of rubber particles from waste tires as a replacement for traditional concrete aggregates. Concrete created using this approach, or rubber concrete, not only addresses black pollution from industrial waste residue but also reduces over-exploitation of sandstone resources. Moreover, compared to conventional concrete, rubber concrete offers superior performance in sound absorption^4^, heat insulation^5^, impermeability^6^, frost resistance^7^, and shock absorption^8^. However, the addition of rubber particles to concrete does come with a trade-off, it reduces the compressive and flexural strength of concrete^9^. To mitigate this negative impact, research has focused on finding additives that can enhance the mechanical properties of rubber concrete.

Silica fume has a relatively small particle size, and the potential to reduce the porosity and increase the density and thus compressive strength of concrete matrixes. Silica fume further exhibits strong pozzolanic activity, reacting with Ca(OH)_2_ generated by cement hydration to form a C–S–H gel. This process effectively repairs surface cracks that can result from the use of coarse aggregates and enhances the interfacial bonding strength (or adhesion) in concrete^10^. Consequently, numerous scholars have conducted extensive research examining the properties of silica fume-reinforced concrete. Salehi et al.^11^ examined the relationship between varying silica fume content and concrete failure modes. It was observed that as silica fume content increased from 0 to 5%, the initial fracture energy of the concrete matrix increased by 19%, while fracture toughness increased by 14%. Zhang et al.^12^ further established that when other conditions are held constant, silica fume introduction can improve both porosity and micro-defects in concrete. Further notable, the strength of the interfacial transition zone is enhanced in silica fume-reinforced concrete.

Polypropylene fiber addition can enhance the bearing capacity and toughness of concrete by effectively inhibiting the formation and expansion of internal cracks. However, it has little effect on concrete’s compressive strength. Therefore, improving the performance of polypropylene fiber-modified concrete to achieve both high strength and high toughness has become a pivotal research focus. Notably, Yoo et al.^13^ initiated the exploration of polypropylene fiber-reinforced concrete and found that the integration of polypropylene and other synthetic fibers contributed to the enhancement of cement matrix strength, particularly in terms of material impact resistance. Makita et al.^14^ further established that polypropylene fiber significantly enhances the impact resistance and residual ductility of concrete. But Wang et al.^15^ study conducted with polypropylene fiber volume content (0%, 0.05%, 0.10%, 0.15%) as the primary variable indicated that polypropylene fiber did not significantly improve the compressive strength of concrete, although it did enhance the final failure state.

Numerous intriguing results have been reported regarding improving the compressive and flexural strength of rubber concrete through various means including the addition of additives. Of note, Agrawal et al.^16^ found the use of NaOH pretreatment methods with 20 mm long rubber fibers enhanced the tensile strength of rubber concrete by approximately 10%. Youssf et al.^17^ discovered that 2 h of pre-treatment of the rubber at 200 °C played a crucial role in improving impact resistance of rubber concrete when substituting 40% to 80% of traditional concrete aggregate content with heat-treated rubber. Further, they found the utilization of water magnetized for 24 h increased the compressive strength by 50%. Abdal et al.^18^ found employing two types of fibers enhanced the mechanical properties of rubber concrete at a fiber dosage of 15 kg/m^3^. However, these novel methods are associated with high costs and complex processes. Moreover, although Su et al.^19^ mitigated the strength reduction of rubber concrete under various high temperatures through the addition of basalt and polypropylene fibers, they did not investigate the microstructure of rubber concrete under the influence of fibers to identify the strengthening mechanism.

At present, numerous studies have been conducted in China to investigate the impact of single-doped silica fume or polypropylene fiber on the mechanical properties of rubber concrete. However, research combining the advantages of both dopants and investigating their comprehensive mechanical properties remains limited. To address this gap, this paper aims to systematically investigate rubber concrete and its silica fume and polypropylene fiber modified specimens. In particular, it focuses on studying the static and dynamic mechanical properties of double-doped silica fume and polypropylene fiber modified rubber concrete. Furthermore, it analyzes the strengthening mechanism of silica fume and polypropylene fiber at the micro level. Ultimately, this study aims to provide a theoretical basis for the production of high-strength, high-toughness rubber concrete.

## Test

### Materials

The P.O 42.5 grade normal Portland cement serves as the binder. The coarse aggregate consists of gravel with a particle size ranging from 5 to 15 mm, while the fine aggregate is river sand with an apparent density of 2410 kg/m^3^ and a fineness modulus of 2.6. The rubber particles have a particle size of 20 mesh and an apparent density of 950 kg/m^3^. The polypropylene fiber measures 12 mm in length and has a density of 910 kg/m^3^. The silica fume is a white powder containing approximately 98% silicon with an average particle size of 0.1–0.2 μm, and the specific surface area was approximately 30.6 m^2^/g. In addition, the silica fume also contains 0.4% Al_2_O_3_ and 0.6% CaO. The water reducing agent is a polycarboxylate superplasticizer, and regular tap water is used.

### Mix proportion and specimens preparation

Utilizing C40 concrete as a base material, in accordance with JGJ55-2011. Many studies have shown that when the rubber content reaches 20%, the strength of concrete will significantly decrease, but it still falls within an acceptable range^20,21^. In addition, it is only when there is a notable decrease in the strength of rubber concrete that the use of silica fume and fiber reinforcement becomes more meaningful. Therefore, in this study, 20 mesh rubber particles were used to replace 20% of the fine aggregate in equal volume^22^.

The study also incorporated silica fume replacing 0% and 10% of the cement in equal volume. Polypropylene fiber was mixed into the concrete with volume fractions of 0%, 0.05%, 0.1%, 0.15%, and 0.2% for mechanical property testing and microscopic examination^23^. The abbreviations C and SC represent normal concrete and silica fume concrete, respectively, while PRC and SPRC represent polypropylene fiber rubber concrete and silica fume polypropylene fiber rubber concrete, respectively. The test encompassed a total of 12 groups, as shown in Table 1.

**Table 1:** Concrete mix ratio (kg/m^3^).

Numbered	Silica fume	Water	Cement	Fine aggregate	Coarse aggregate	Rubber	Fiber	Water reducer
C	0	160	435	470.4	1212	0	0	2
SC	43.5	160	435	470.4	1212	0	0	2
PRC-1	0	160	435	470.4	1212	46.36	0	2
PRC-2	0	160	435	470.4	1212	46.36	0.455	2
PRC-3	0	160	435	470.4	1212	46.36	0.91	2
PRC-4	0	160	435	470.4	1212	46.36	1.365	2
PRC-5	0	160	435	470.4	1212	46.36	1.82	2
SPRC-1	43.5	160	391.5	470.4	1212	46.36	0	2
SPRC-2	43.5	160	391.5	470.4	1212	46.36	0.455	2
SPRC-3	43.5	160	391.5	470.4	1212	46.36	0.91	2
SPRC-4	43.5	160	391.5	470.4	1212	46.36	1.365	2
SPRC-5	43.5	160	391.5	470.4	1212	46.36	1.82	2

To conduct tests on compressive, splitting tensile, flexural, and impact performance, the following specimens were prepared: The cube specimen with a size of 100 mm, cuboid specimens measuring 400 mm in length, 100 mm in width, and 100 mm in height, and a cylindrical specimen with a diameter of 50 mm and a height of 25 mm. Each group consisted of twelve specimens, resulting in a total of 12 groups.

### Experimental design

#### Static test

**Figure 1:**
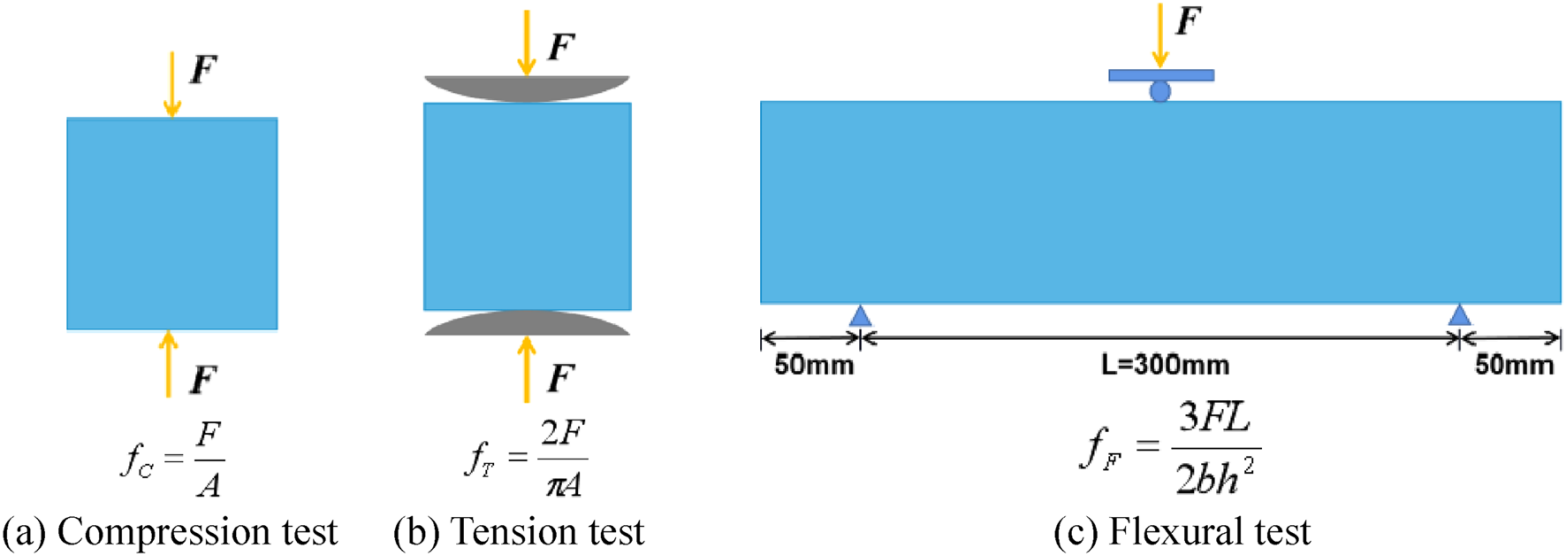
The loading method and strength calculation diagram. (**a**) Compression test, (**b**) Tension test, (**c**) Flexural test.

This experiment utilizes the WAW-1000 electro-hydraulic servo universal testing machine to conduct compression, tension, and flexural strength tests on rubber concrete and its modified specimens. The loading is stopped when the stress–strain curve begins to stabilize, with loading speeds of 1 mm/min, 1 mm/min, and 0.5 mm/min, respectively. The loading method and strength calculation diagram are shown in Fig. 1. Among them, the static compression, tension, and flexural strength tests are conducted to analyze the process of slow deformation in structures such as buildings, tunnels, coal mines, and so on.

#### Dynamic test

**Figure 2:**
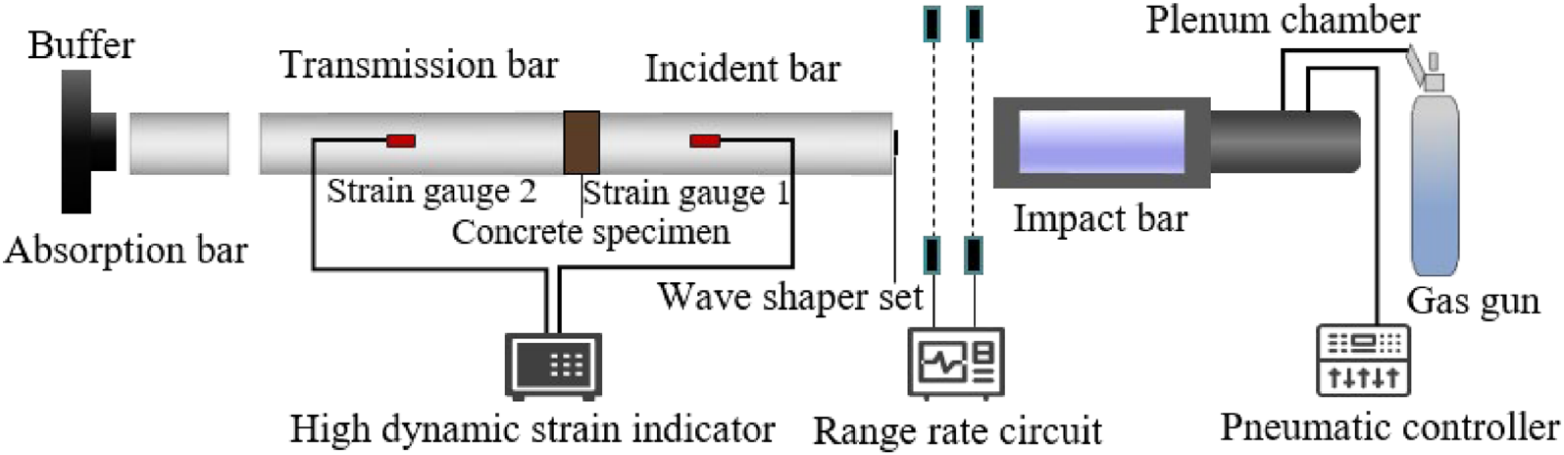
The schematic diagram of the SHPB test device.

Aiming at the problem of dynamic damage caused by rock burst, earthquake and explosion, the SHPB (Split Hopkinson Pressure Bar) device was used to conduct the dynamic compressive tests on rubber concrete specimens with varying polypropylene fiber contents (0%, 0.05%, 0.1%, 0.15%, 0.2%) and different silica fume contents (0%, 10%), all under a consistent air pressure of 0.3 MPa (The strain rate is 34 s^−1^). The objective was to analyze the compressive strength and failure mode characteristics of silica fume fiber rubber concrete under impact conditions. The test device and schematic diagram are shown in Fig. 2. The rod is a high alloy steel with an elastic modulus of 210GPa and a wave velocity of 5190 m/s. Prior to testing, precise adjustments were made to ensure the impact bar, incident bar, and transmission bar were aligned on the same horizontal plane. To minimize end face friction, Vaseline was applied to both the specimen's surface and the bars^24,25^. During the test, high-pressure nitrogen propelled the punch at a specific velocity against the incident bar, converting its kinetic energy into incident energy. This energy, transmitted to the specimen in the form of waves, resulted in partial conversion into reflected energy stored within the reflected wave. Additionally, a portion was stored in the transmitted wave and absorbed by the energy-absorbing device, while the remainder was predominantly absorbed by the specimen, contributing to the formation of new cracks^26^. Test data was gathered by collecting pulse signals through strain gauges positioned on both the incident and transmission bars. These pulse signals were then converted into strain signals by the strain testing system. From these readings, stress and strain values were calculated using Formula (1).

1 $$\left\{ \begin{gathered} \sigma (t) = \frac{A}{{A_{s} }}E\varepsilon_{t} \hfill \\ \varepsilon (t) = \frac{C}{{L_{s} }}\int_{0}^{t} {(\varepsilon_{i} - \varepsilon_{r} - \varepsilon_{t} )} dt \hfill \\ \end{gathered} \right.$$

In the formula, *σ* and *ε* represent the stress and strain of the specimen, respectively. *E*, *A* and *C* represent the elastic modulus, cross-sectional area, and wave velocity of the compression bar, respectively. *A*_*s*_ and *L*_*s*_ stand for the initial cross-sectional area and initial length of the specimen, respectively. *ε*_*i*_, *ε*_*r*_ and *ε*_*t*_ represent the incident strain, reflection strain, and transmission strain, respectively.

**Figure 3:**
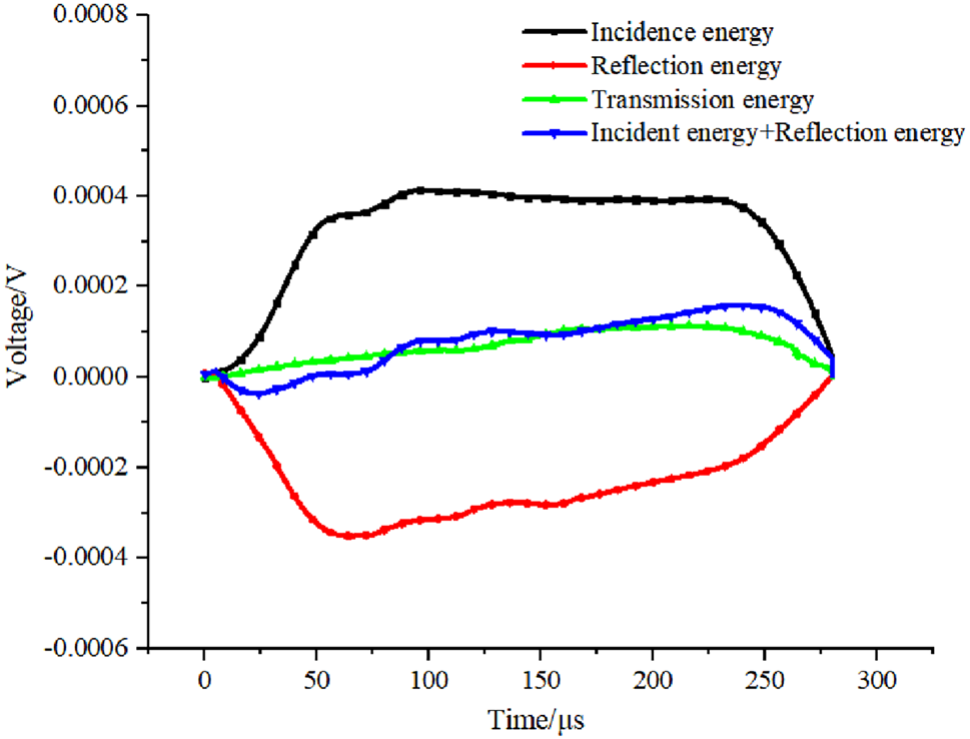
Dynamic stress equilibrium.

The dynamic compressive strength of the specimen is tested with the Split Hopkinson Pressure Bar (SHPB) device. During the impact test loading process, whether the stress at both ends of the specimen can achieve balance determines the reliability of the test results^27^, so it is necessary to verify the stress balance. The degree of dispersion between the sum of the incident wave and the reflected wave and the transmitted wave curve can reflect the stress balance state of the combined specimen during the loading process. As can be seen from Fig. 3, the two curves fit well, so the stress balance at both ends of the specimen can be satisfied.

#### Microstructure

In scanning electron microscopy (SEM) test, scanning electron beam was used to excite physical signals from the sample surface and modulate the imaging, so that the microscopic three-dimensional morphology of the sample can be obtained. The concrete in the core area of compressive test specimen was cut into slices with the size of 6 mm × 6 mm × 4 mm. After drying the slices, the microstructure was studied by using FlexSEM1000 scanning electron microscope produced by Hitachi, Japan.

## Test results and analysis

### Compressive strength test

#### Stress–strain curves

**Figure 4:**
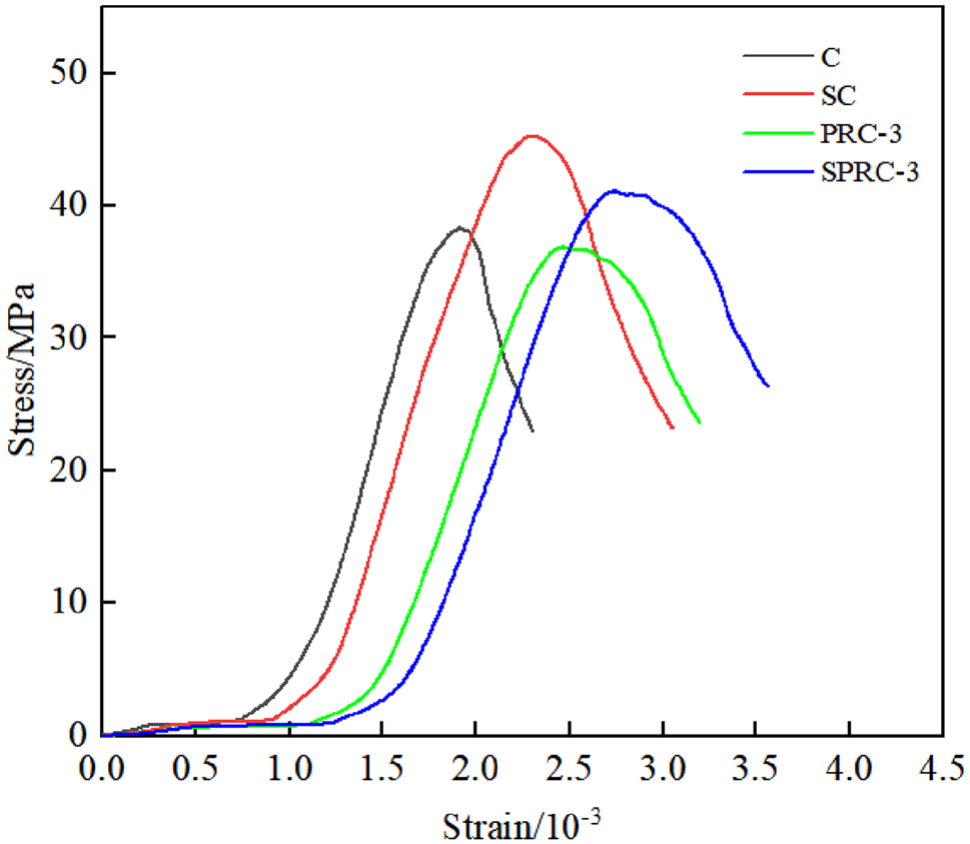
Stress–strain curves of compressive test.

Concrete members are primarily subjected to compression, making compressive strength the most fundamental mechanical performance indicator of concrete. Figure 4 depicts the stress–strain curves for normal concrete (C), silica fume concrete (SC), polypropylene fiber rubber concrete (PRC), and silica fume polypropylene fiber-modified rubber concrete (SPRC) in a compressive strength test. It is evident that silica fume and fiber exert distinct effects on the failure process of concrete.

**Figure 5:**
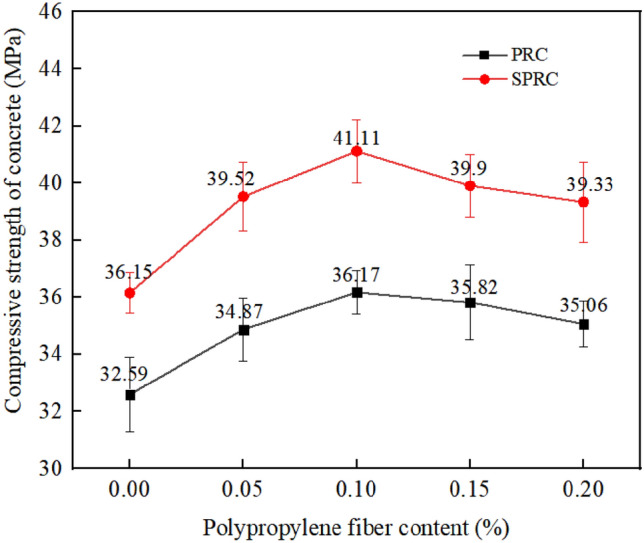
Compressive strength of concrete with different fiber content.

The impact of various fiber contents on the compressive strength of concrete is presented in Fig. 5. The cube compressive strength of normal concrete C, silica fume concrete SC, rubber concrete PRC-1, polypropylene fiber rubber concrete PRC-3 and mixed silica fume, polypropylene fiber modified rubber concrete SPRC-3 are 38.32 MPa, 45.23 MPa, 32.59 MPa, 36.17 MPa and 41.11 MPa, respectively. Notably, the compressive strength of silica fume concrete SC is 18% higher than that of normal concrete after the addition of 10% silica fume. This increase is attributed to the small particle size of silica fume, which effectively fills the gaps within normal concrete and enhances the compactness of its internal structure, thereby elevating the compressive strength of the specimen^28,29^.

The compressive strength of the specimen decreased significantly with the incorporation of rubber particles, registering a 15% reduction compared to normal concrete. This decrease can be attributed to the inherent characteristics of rubber particles, which possess low strength and high elasticity^30^. Additionally, the adhesion between the rubber particles and the concrete matrix was poor, when rubber particles are mixed with concrete as fine aggregate, the specimen exhibits reduced compressive strength^31,32^. However, upon the addition of 0.1% fiber, the compressive strength of specimen PRC-3 increased by 11% compared to PRC-1. This observation indicates that the randomly distributed fiber plays a reinforcing role, effectively hindering the expansion of internal cracks and thereby enhancing the strength of the specimen^33,34^.

Figure 5 evident that the addition of fiber enhances the compressive strength of concrete. When no fiber is present (0% fiber content), the compressive strength of PRC-1 is 32.59 MPa. However, with increasing fiber content of 0.05%, 0.1%, 0.15%, and 0.2%, the compressive strength of the specimens increases by 7.00%, 10.98%, 9.91%, and 7.58%, respectively. It is noteworthy that the compressive strength initially increases with the fiber content but subsequently decreases, beginning to decline when the fiber content exceeds 0.10%. This trend can be attributed to two primary reasons. Firstly, excessive fibers cannot be evenly distributed within the concrete, resulting in clusters of fibers. These clusters create larger gaps within the concrete structure, leading to stress concentration and damage at these weak points during compression^35^. Secondly, the larger gaps reduce the overall compactness of the concrete, thus reducing its compressive strength^36^. Under identical fiber contents, the compressive strength of SPRC concrete is significantly higher than that of PRC after the addition of silica fume. Notably, when the polypropylene fiber content is 0.1%, the compressive strength of the specimen increases by 13.99%. This improvement can be attributed to the Pozzolanic activity of silica fume and the filling effect of fine particles, both of which enhance the compactness of the specimen's structure^37^.

#### Failure modes

**Figure 6:**
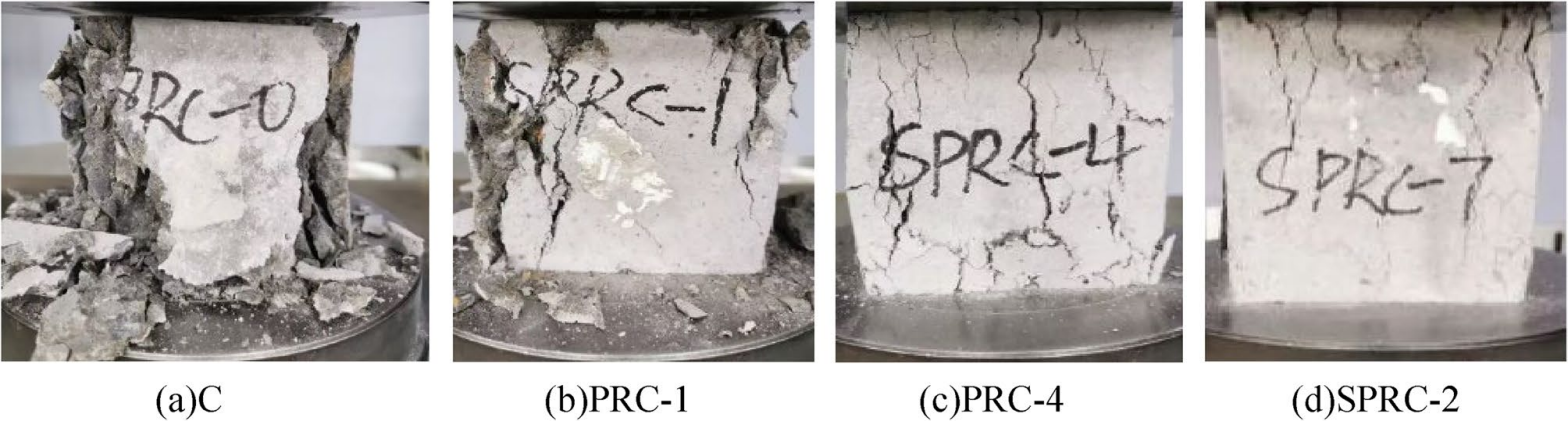
Failure form of concrete compressive strength test. (**a**) C, (**b**) PRC-1, (**c**) PRC-4, (**d**) SPRC-2.

In the compressive strength test of concrete, a fine crack parallel to the stress direction emerged in the middle of the surface of the normal concrete specimen. As the stress intensified, the specimen expanded towards the unstressed surface, and some of the concrete on the surface began to detach. Examination of the detachment site revealed a significant number of concrete aggregates exposed, indicating more prominent internal cracks. As the load increased, specimen deformation grew, the number of cracks multiplied, and the width expanded. When the load exceeded the ultimate limit, the specimen emitted a violent noise, with serious concrete aggregate detachment in a wedge-shaped pattern. This indicated a clear brittle failure, as exemplified in Fig. 6a. In contrast, when rubber concrete was subject to destruction, the sound generated was much lower than that of normal concrete. Although specimen detachment was not significant, when pressure reached the ultimate load, its bearing capacity plummeted. The number of cracks on the rubber concrete specimen's surface correspondingly increased, as displayed in Fig. 6b.

By introducing fiber into rubber concrete, the deformation and surface cracks of the specimen are significantly improved. The specimen's anti-deformation capacity is noticeably enhanced, with a reduced number of surface cracks and significantly narrower widths. As the load increases, the specimen's surface transitions from the previously described wide crack to multiple fine cracks. When the ultimate load is reached, the surface cracks do not penetrate, reducing the area of concrete block detachment and enhancing specimen integrity, as displayed in Fig. 6c. When silica fume and fiber are mixed, cracks begin to appear in the upper part of the specimen. As the load increases, the number of cracks gradually increases and extends to the lower part of the specimen until the ultimate load is reached. Surface concrete debris detaches to a lesser extent, as shown in Fig. 6d.

The distinct failure modes of the aforementioned concrete specimens are primarily attributed to the introduction of rubber particles which exhibit low strength but high elasticity into the concrete. This enhances the specimens' toughness. The fibers, which are randomly distributed in three dimensions within the concrete, form a network structure that impedes crack expansion and improves both the strength and ductility of the specimens^33^. Additionally, silica fume contributes its pozzolanic activity and fine particle filling effect, enhancing the structural compactness of the specimen^32,37^.

### Splitting tensile strength test

#### Test results and analysis

**Figure 7:**
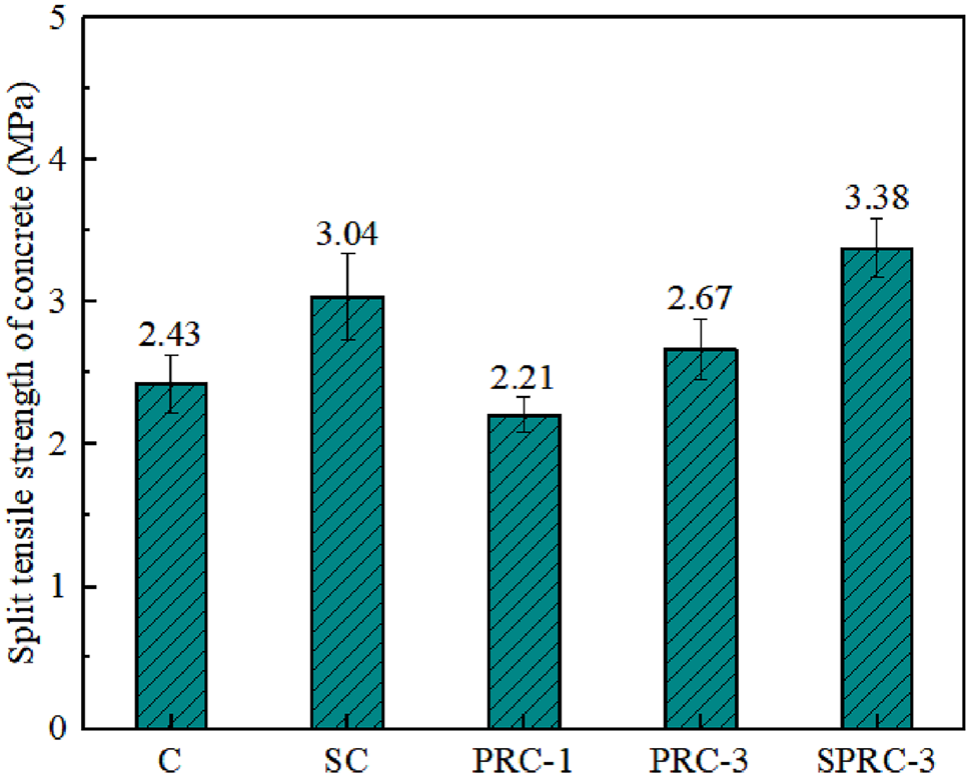
Split tensile strength of different types of concrete specimens.

The splitting tensile strength of concrete is presented in Fig. 7. The specimens C, SC, PRC-1, PRC-3, and SPRC-3 exhibit splitting tensile strengths of 2.43 MPa, 3.04 MPa, 2.21 MPa, 2.67 MPa, and 3.38 MPa, respectively. Analysis reveals that the addition of silica fume enhances the splitting tensile strength of the specimen. Notably, the splitting tensile strength of SC is 25.1% higher than that of specimen C. However, incorporating rubber particles into the concrete reduces the splitting tensile strength of PRC-1 by 9.05% compared to specimen C. This decrease is primarily due to the increased presence of weak bonding surfaces and weak points within the concrete, reducing the effective area available for bearing tensile stress within the matrix and thus diminishing the splitting tensile strength of the specimen. The addition of fiber significantly improves the splitting tensile strength of the specimen. On this basis, when silica fume is added to specimens containing fiber, the splitting tensile strength is further enhanced. In particular, the specimens SC, PRC-3, and SPRC-3 exhibit splitting tensile strengths that are 9.88%, 39.09%, and 39.09% higher than those of normal concrete C, respectively. These findings indicate that compared to single-doped fibers, the combination of silica fume and fibers is more effective in enhancing the splitting tensile properties of the specimens.

**Figure 8:**
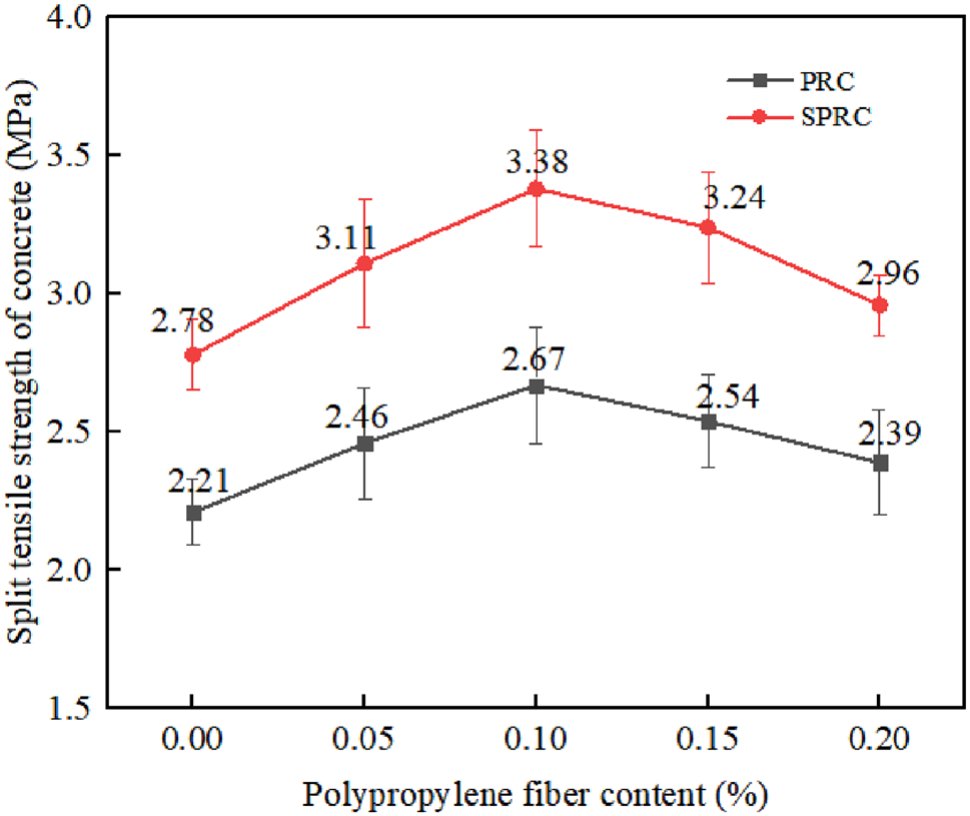
Split tensile strength of concrete with different fiber content.

Figure 8 shows the impact of varying fiber content on the splitting tensile strength of concrete. As the figure reveals, the splitting tensile strength of rubber concrete PRC-1 without fiber is 2.21 MPa. However, when 0.05%, 0.1%, 0.15%, and 0.2% fibers are introduced, the splitting tensile strength of the cube increases by 11.31%, 20.81%, 14.93%, and 8.14% respectively. This suggests that the inclusion of a specific amount of fiber significantly enhances the splitting tensile strength of rubber concrete, among them, when the fiber content is 0.1%, the improvement effect is the most obvious. However, beyond this content, the splitting tensile strength of each group of rubber concrete begins to decrease. This decline may be attributed to excessive fiber content in rubber concrete, where fiber agglomeration fails to provide reinforcement^35^.

The addition of silica fume to specimen PRC has a significant impact on SPRC's splitting tensile strength. On a macroscopic level, fibers are randomly distributed within the concrete, immediately blocking cracks as they encounter adjacent fibers. This prevents cracks from expanding and extending, thereby enhancing the splitting tensile strength of concrete. On a microscopic scale, the introduction of silica fume into the matrix reacts to form a new gel, improving the weak interface introduced by rubber’s addition. Additionally, the filling effect of fine particles reduces the number of pores in the specimen and enhances the compactness of the rubber concrete specimen, greatly enhancing its splitting tensile strength^38^.

#### Failure modes

**Figure 9:**
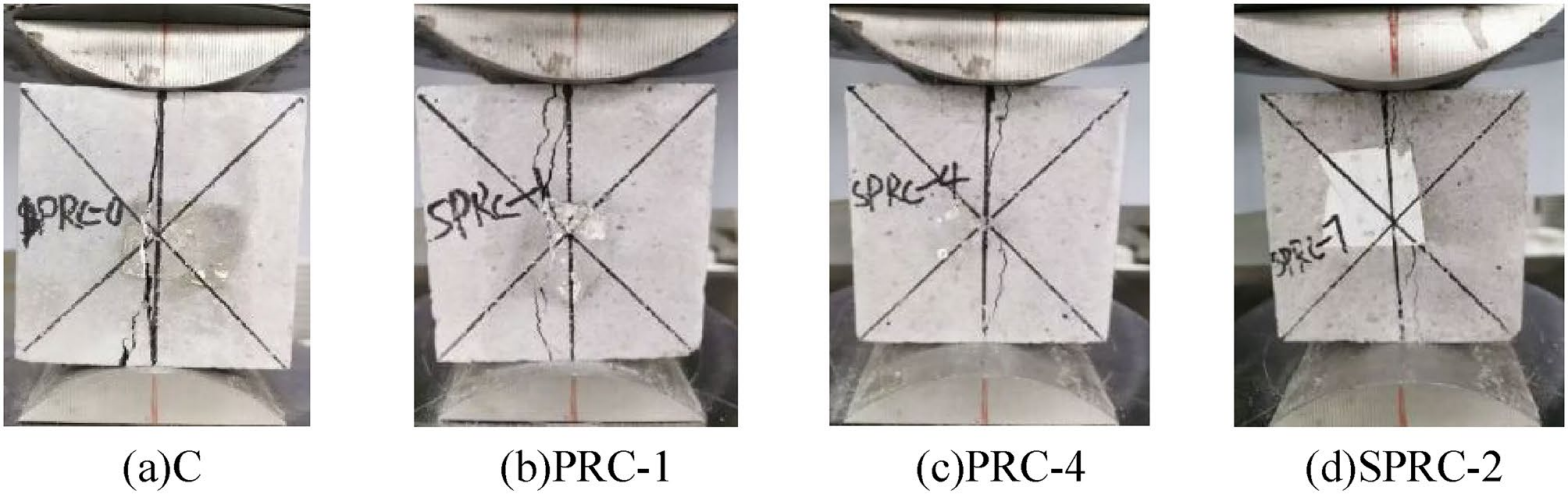
Failure pattern of concrete splitting tensile strength test. (**a**) C, (**b**) PRC-1, (**c**) PRC-4, (**d**) SPRC-2.

Figure 9 shows the failure mode of concrete splitting tensile test. After the test commenced, specimen C exhibited cracks on its surface quickly under the applied load. These cracks rapidly propagated vertically, ultimately traversing the entire specimen. At this point, a loud sound of damage was heard, and the load promptly decreased to zero, resulting in a wide crack visible in Fig. 9a. Upon the inclusion of rubber particles, the specimen's damage sound was noticeably reduced compared to normal concrete. The specimen's surface cracks were numerous and smaller, as shown in Fig. 9b. This phenomenon occurred because the rubber particles were evenly distributed within the concrete, mitigating stress concentration at the crack tips. During loading, these particles converted macro cracks in the matrix into numerous micro cracks, thereby reducing stress concentrations within the concrete's pores.

When polypropylene fiber was added, numerous microcracks appeared on the specimen's surface during the loading process. As the load increased, the length and width of these cracks expanded. When peak stress was reached, penetrating cracks formed, yet the specimen retained its integrity, as seen in Fig. 9c. This indicates that adding polypropylene fiber enhances the splitting tensile strength of rubber concrete. When silica fume and fiber were mixed, the specimen exhibited more cracks on its surface with narrower widths. The test duration was longer, and no visible through cracks appeared until failure^39^, as shown in Fig. 9d.

#### Tension–compression ratio

**Figure 10:**
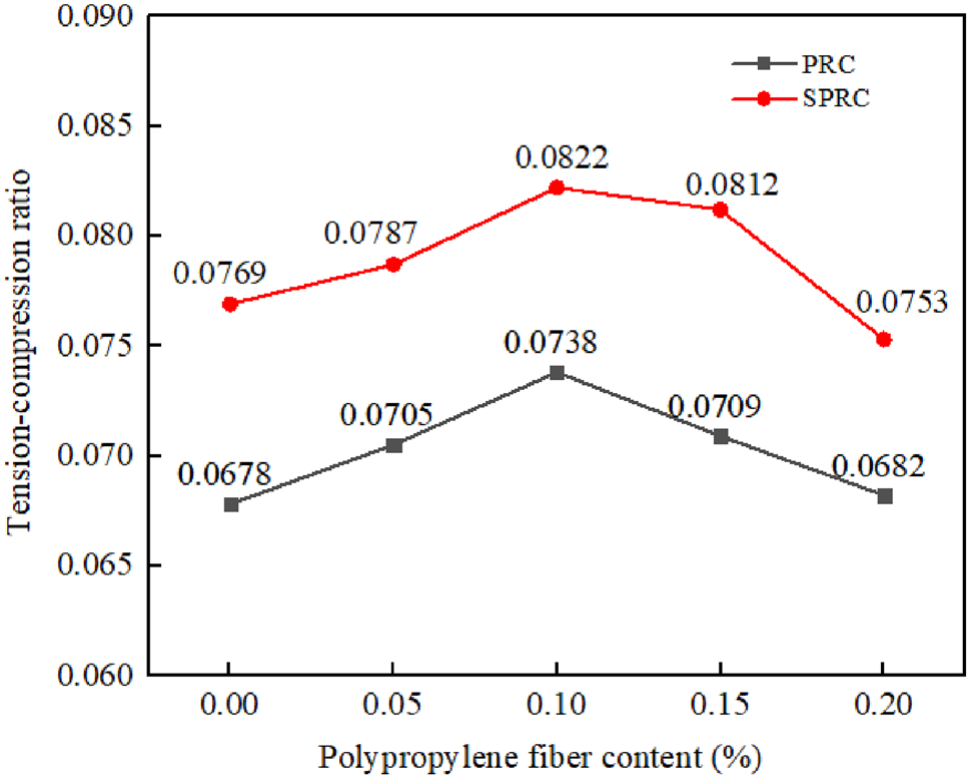
Tension- compression ratio of concrete with different fiber content.

The ratio of splitting tensile strength to compressive strength serves as a crucial metric for assessing the brittleness of concrete materials^40^. A higher tension–compression ratio indicates reduced brittleness and enhanced toughness in the specimen. Figure 10 illustrates the variation curve of the concrete tension–compression ratio as the varying content of polypropylene fiber. Notably, the tension–compression ratio for PRC-1 is 0.0678, while that of C is 0.0634. The incorporation of rubber particles does improve the brittleness of concrete to some extent, albeit with a reduction in strength. Building on PRC-1, the addition of 0.05%, 0.1%, 0.15%, and 0.2% polypropylene fibers results in tension–compression ratios of 0.0705, 0.0738, 0.0709, and 0.0682, respectively. It shows that the introduction of fibers not only bolsters the strength of rubber concrete but also enhances its toughness. When compared to PRC, the tension–compression ratio of SPRC experiences further improvement. This finding suggests that the combination of silica fume and polypropylene fiber is more effective in elevating the tension–compression ratio of rubber concrete than the use of polypropylene fiber alone^41,42^.

### Flexural strength test

#### Test results and analysis

**Figure 11:**
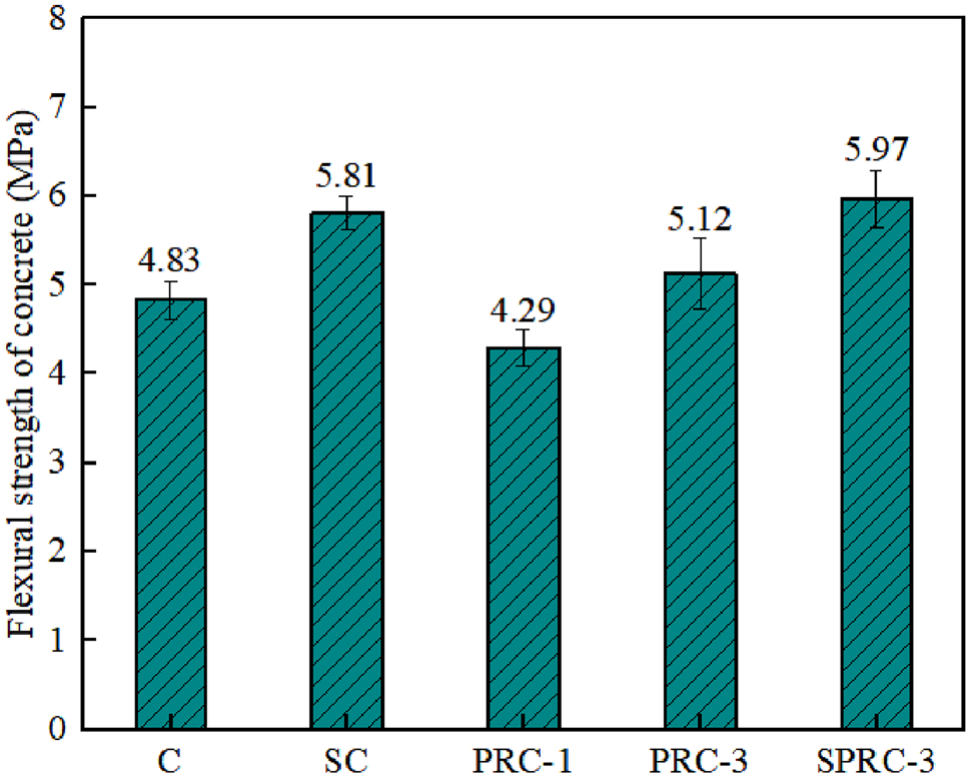
Flexural strength of different types of concrete.

The flexural strength values for various types of concrete are presented in Fig. 11. Specifically, the flexural strengths for specimens C, SC, PRC-1, PRC-3, and SPRC-3 measure 4.83 MPa, 5.81 MPa, 4.29 MPa, 5.12 MPa, and 5.97 MPa, respectively. Notably, the addition of silica fume alone enhances the flexural strength of normal concrete. In fact, the flexural strength of SC is 20.29% higher than that of C, likely due to silica fume's filling effect, which improves concrete compactness. However, when rubber particles are introduced into normal concrete, as seen in PRC-1, the flexural strength decreases by 11.18%. This decrease is attributed to the physical and mechanical properties of rubber, which create weaker bonding points within the concrete and reduce its effective bending area^43^.

Fortunately, the addition of fibers to rubber concrete significantly improves flexural strength, as seen in the PRC-3 specimen. Furthermore, the inclusion of silica fume on this basis further elevates the flexural strength. Compared to PRC-1, the flexural strengths of PRC-3 and SPRC-3 specimens increase by 19.34% and 39.16%, respectively. These findings underscore the effectiveness of silica fume in enhancing the flexural strength of concrete^44^.

**Figure 12:**
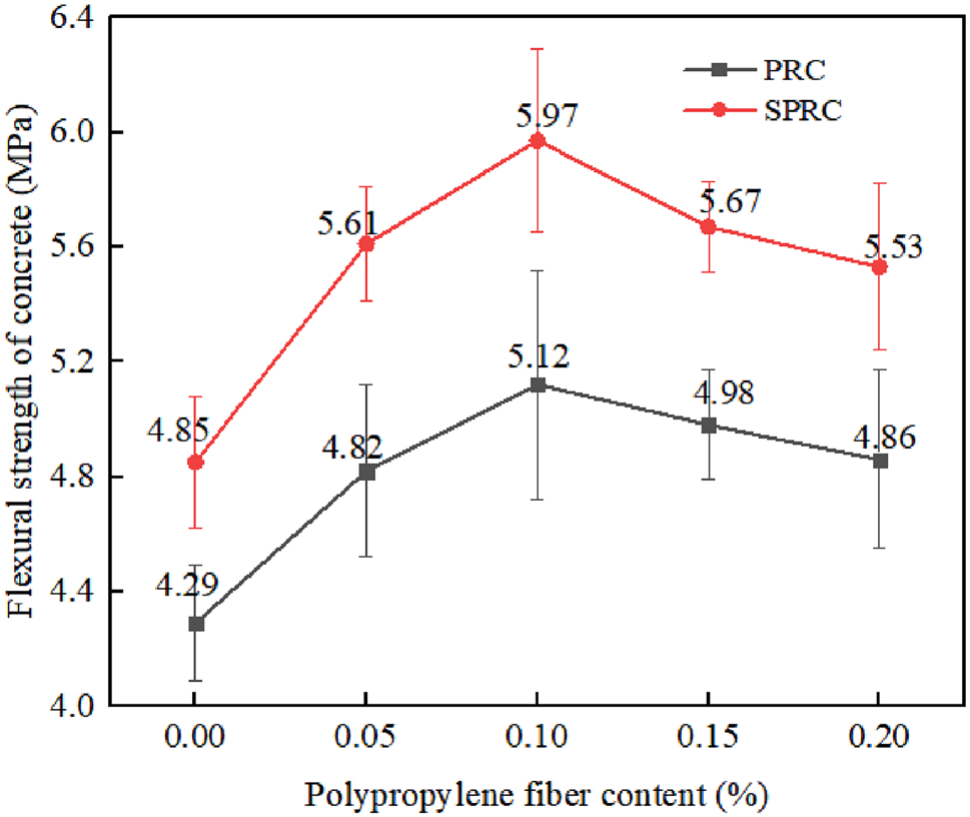
Flexural strength of concrete with different fiber dosages.

Figure 12 demonstrates the impact of various polypropylene fiber contents on the flexural strength of concrete. As the fiber content increases from 0.05 to 0.1%, the flexural strength of PRC specimens initially increases, increased by 12.35% and 19.35% respectively. However, further increases in fiber content to 0.15% and 0.2% result in a reduction of the flexural strength enhancement effect, achieving only 16.08% and 13.29% growth, respectively. This suggests that an excessive fiber content can negate the positive effects on PRC's flexural strength. When compared to PRC, the flexural strengths of SPRC-1, SPRC-2, SPRC-3, SPRC-4, and SPRC-5 exhibit greater improvement under identical fiber content conditions. Their flexural strengths increase by 13.05%, 16.39%, 16.60%, 13.86%, and 13.79%, respectively. This underscores the remarkable enhancing effect of silica fume on the performance of the interfacial transition zone, matrix density, and bonding between aggregate, cement matrix, and fiber^45^.

#### Failure modes

**Figure 13:**
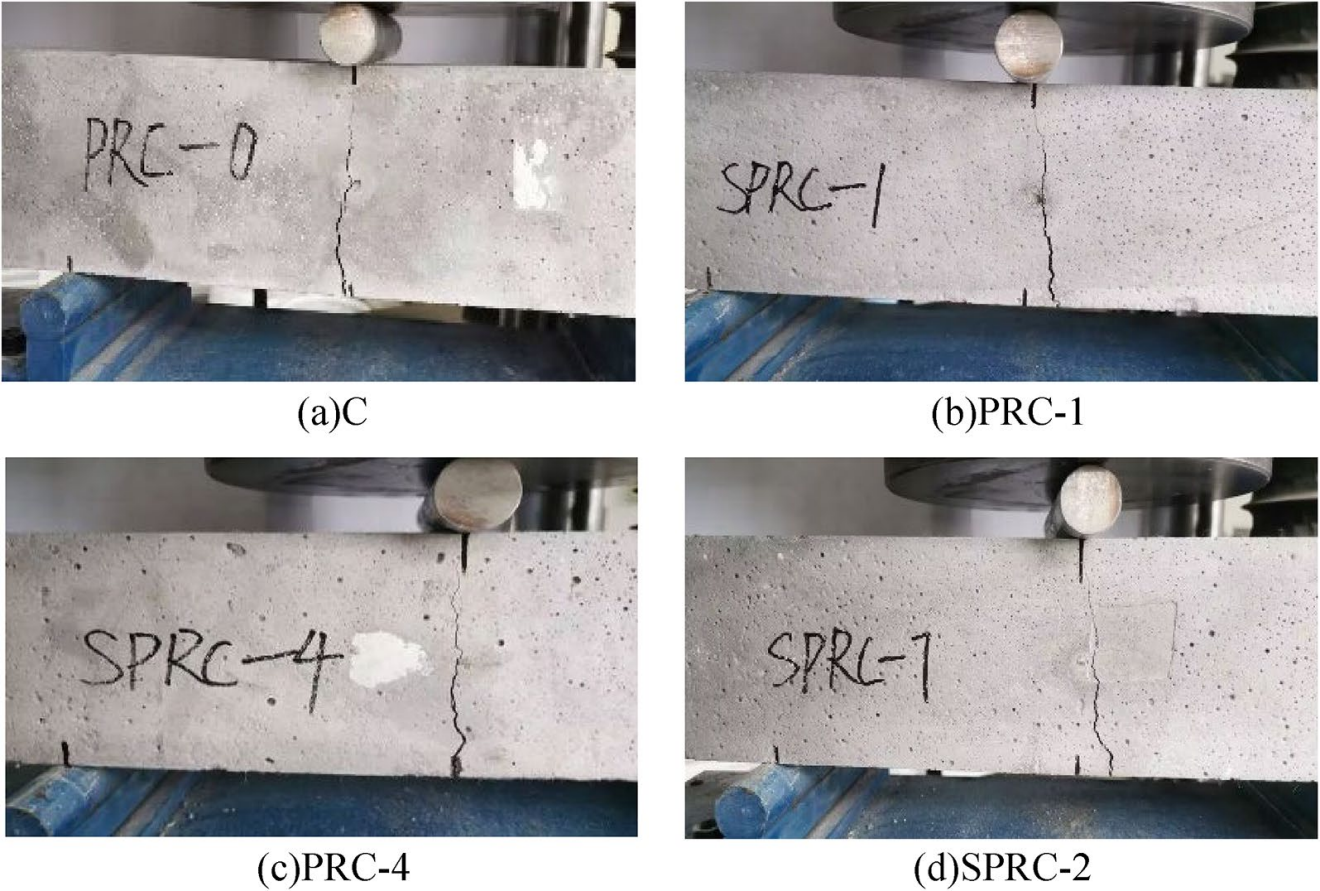
Failure form of concrete flexural strength test. (**a**) C, (**b**) PRC-1, (**c**) PRC-4, (**d**) SPRC-2.

The failure mode in the flexural test of concrete is presented in Fig. 13. As the load is gradually applied, specimen C emits a noticeable sound, yet there are no visible changes on its surface. As the load approaches the ultimate load, the sound generated by the specimen's force gradually intensifies. When the ultimate load is reached, the load decreases sharply along with a loud and destructive sound, indicating a brittle fracture, as shown in Fig. 13a. When rubber particles are added to normal concrete, the sound emitted by PRC-1 diminishes, but the number of cracks increases, as displayed in Fig. 13b.

With the addition of fibers, PRC-3 exhibits numerous small cracks at the bottom early in the test, which then expand to the upper part. The specimen maintains ductility until its ultimate failure, as seen in Fig. 13c. Upon incorporating silica fume, the specimen initially shows no cracks. However, as stress increases, cracks appear at the bottom and gradually expand upward. When peak stress is reached, the cracks do not penetrate the specimen, indicating a ductile failure, as shown in Fig. 13d.

#### Flexural-compression ratio

**Figure 14:**
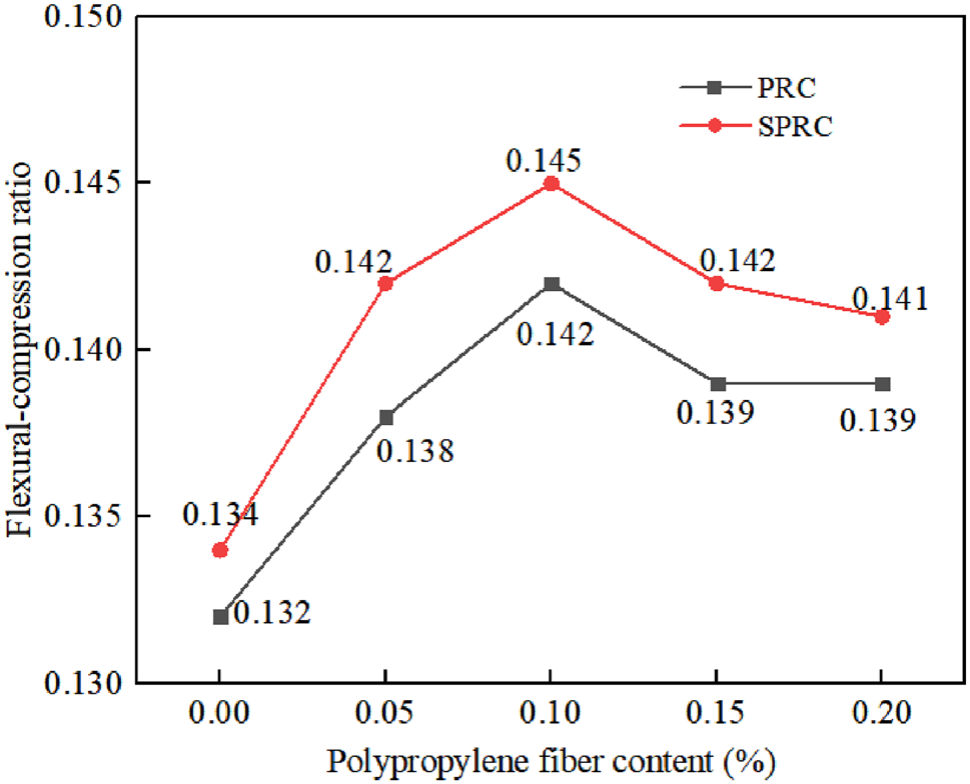
Flexural-compression ratio of concrete with different fiber content.

The ratio of flexural strength to compressive strength serves as a crucial indicator of concrete materials' ductility and crack resistance. A higher flexural-compression ratio signifies lower brittleness and superior toughness^46^. Figure 14 illustrates the flexural-compression ratio's variation with polypropylene fiber content. As the fiber content rises, the flexural-compression ratio initially increases and then subsequently declines. At 0% fiber volume content, the PRC flexural-compression ratio stands at 0.132, while SPRC's is 0.134. When the fiber volume content reaches 0.1%, the maximum PRC flexural-compression ratio jumps to 0.142, a 7.58% increase from its initial value, and SPRC's maximum flexural-compression ratio surges to 0.145, representing an 8.21% increase from its initial value. These findings suggest that polypropylene fiber enhances concrete's ductility and crack resistance. Moreover, under identical fiber content conditions, SPRC's flexural-compression ratio consistently outperforms PRC's. This advantage can be attributed to polypropylene fiber's ability to minimize internal crack formation and enhance concrete's toughness and tensile strength^47^. However, excessive fiber incorporation can undermine concrete's strength, emphasizing the importance of optimal fiber content for enhancing the flexural-compression ratio^48^.

### Dynamic mechanical test

#### Impact test results and analysis

**Figure 15:**
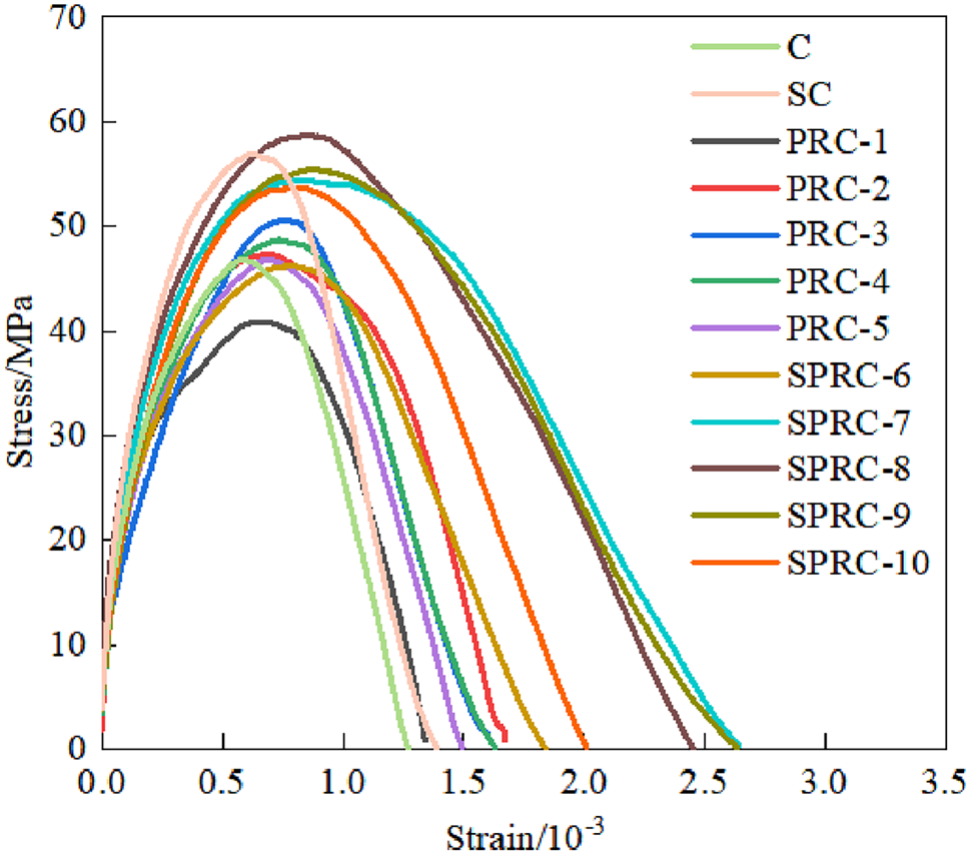
Impact compressive stress–strain curve of concrete with different fiber content.

Figure 15 displays the stress–strain curve of each group of specimens under impact loading.

As Fig. 15 shows, the stress–strain curves share a similar shape. As the fiber volume content increases, the initial elastic modulus, peak stress, and peak strain initially rise and then decline. At a fiber volume content of 0.1%, the three factors reach the maximum values. With the same fiber content, the ascending segment of the stress–strain curve's shape of specimens remains consistent, whereas the descending segment exhibits notable different. The addition of silica fume enhances the initial elastic modulus, peak stress, and peak strain of SPRC compared to PRC^49^. Moreover, the descending section is smoother, indicating that silica fume's modification of PRC results in SPRC achieving superior deformation capacity and ductility performance^50^.

The dynamic compressive strengths of specimens C, SC, PRC-1, PRC-3, and SPRC-3 are 46.93 MPa, 56.85 MPa, 40.84 MPa, 50.6 MPa, and 58.61 MPa, respectively. Notably, the introduction of rubber particles led to a significant decrease in the dynamic compressive strength of the specimen, registering a 12.98% drop compared to C. Conversely, the addition of silica fume boosted the dynamic compressive strength of SC by 21.24% compared to C. When incorporating polypropylene fiber, the dynamic compressive strength of PRC-3 surged by 23.9% compared to PRC-1. Finally, under the combined influence of silica fume and polypropylene fiber, the dynamic compressive strength of SPRC-3 outperformed PRC-3 by 15.8%.

**Figure 16:**
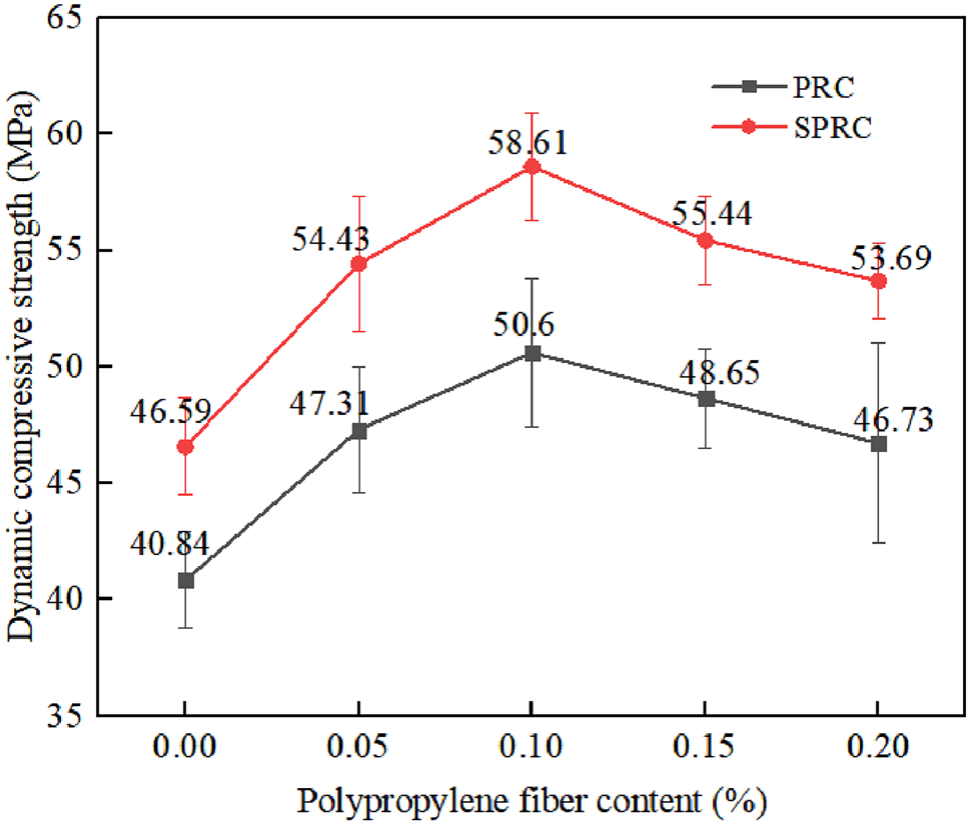
Dynamic compressive strength of concrete with different fiber dosages.

The impact of varying fiber content on the dynamic compressive strength of concrete is further illustrated in Fig. 16. As the fiber volume content increases, the dynamic compressive strength of PRC and SPRC initially rises and then declines. At a fiber volume content of 0.1%, PRC-3 attains a dynamic compressive strength of 50.6 MPa, representing a 23.9% improvement over PRC-1. Similarly, SPRC-3 reaches a maximum dynamic compressive strength of 58.61 MPa, surpassing SPRC-1 by 25.8%. SPRC consistently exhibits a higher dynamic compressive strength than PRC when the fiber volume content is the same. This is primarily due to silica fume's ability to fill and mend cracks and microcracks in concrete, thereby enhancing the performance of the interfacial transition zone^51^.

#### Failure modes

**Figure 17:**
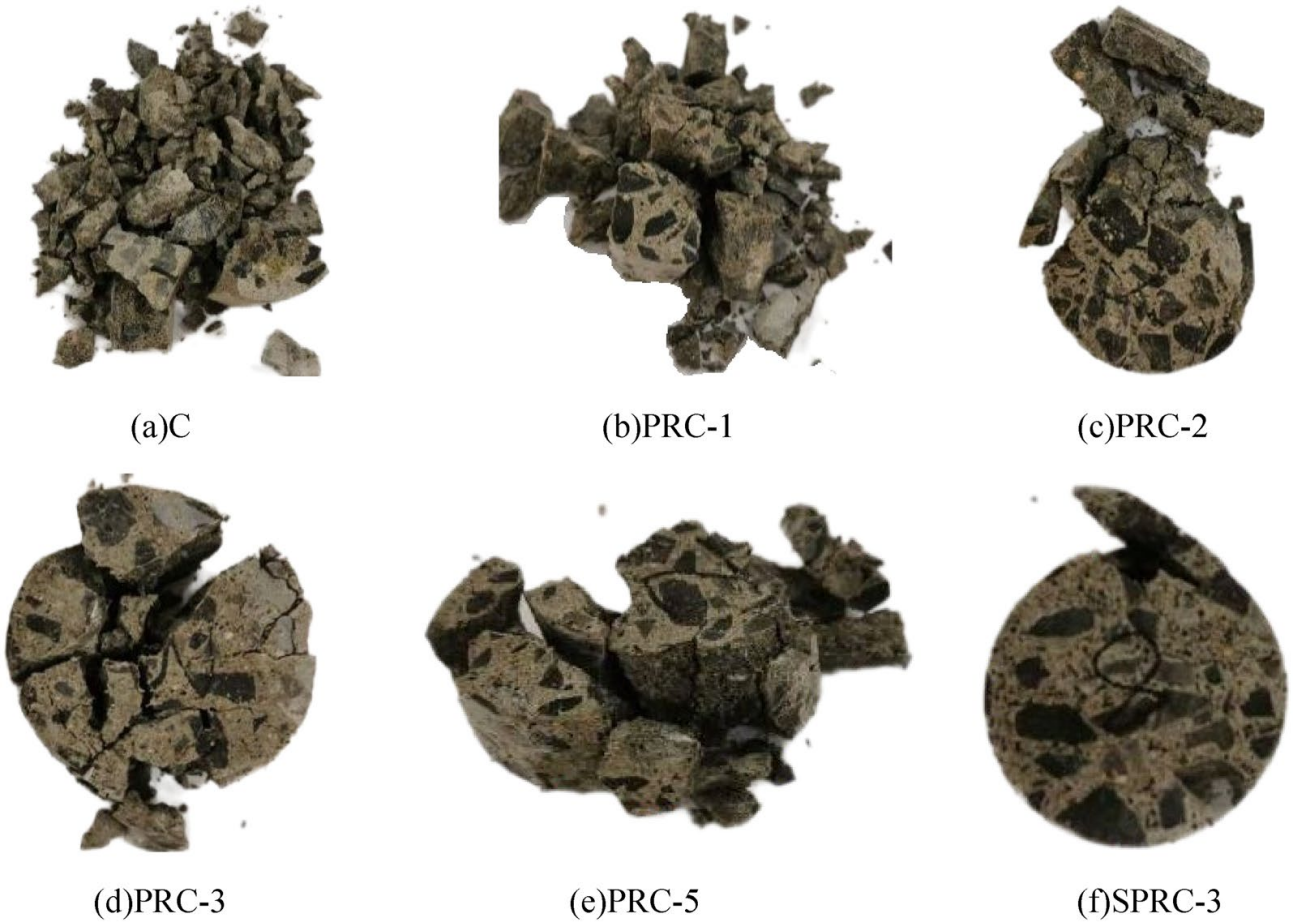
Failure mode of concrete impact. (**a**) C, (**b**) PRC-1, (**c**) PRC-2, (**d**) PRC-3, (**e**) PRC-5, (**f**) SPRC-3.

When exposed to impact load, the degree of rupture and debris characteristics (number and size) of rubber concrete vary depending on the silica fume and polypropylene fiber content. These changes can be observed from various perspectives, as illustrated in Fig. 17

During dynamic compression, as impact kinetic energy is continuously released, cracks propagate until they penetrate the specimen, ultimately leading to its complete destruction. In normal concrete, initial microcracks are predominantly found at the interface between the cement mortar and aggregate. Upon impact, these microcracks do not have sufficient time to expand along the path of least resistance, instead, they propagate directly through the aggregate, resulting in macroscopic cracks^52^. Consequently, normal concrete specimens exhibit crush damage. When rubber particles are added, as seen in Fig. 17b, the excellent elastic deformation characteristics of rubber particles improve the crushing condition of the specimen compared to normal concrete, leading to a more complete failure mode.

When rubber concrete is reinforced with fibers, the fiber's reinforcement capability slows down the crack's propagation speed, allowing it to follow the weakest link within the concrete. Consequently, numerous cracks appear on the specimen's surface, and small amounts of debris detach, as shown in Fig. 17c–e. When the fiber content is low, the load on the crack is reduced, and during the debonding process, the load on the polypropylene fiber is substantial. As the fiber content increases, the tensile capacity of individual fibers gradually diminishes until they can no longer reach their yield limit. When the fiber content exceeds a certain amount, the bonding force between fibers and other factors result in an increase of weak layers within the concrete, leading to a decrease in concrete strength and ultimately leads to the more brittle matrix at the section, as seen in the failure diagram of PRC-5. Therefore, when the polypropylene fiber content is 0.10%, such as in PRC-3, specimen failure is favorable. By adding a certain amount of silica fume, the impact resistance of rubber concrete specimens can be further optimized. This addition significantly reduces the number of fragments resulting from impact damage, as shown in Fig. 17f.

#### Dynamic increase factor

**Figure 18:**
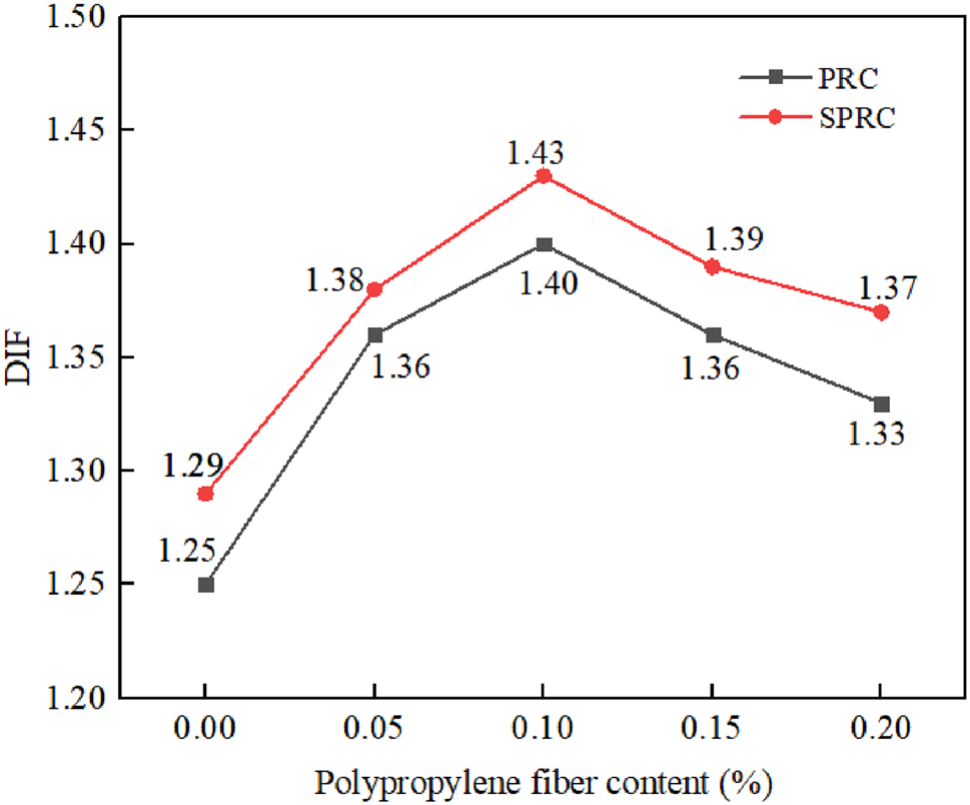
Dynamic increase factor of concrete.

The dynamic increase factor (DIF) is the ratio of dynamic strength to static strength of concrete, serving as a fundamental parameter that characterizes the dynamic properties of materials^53^. Figure 18 shows the relationship between DIF and various fiber contents. As the figure suggests, as fiber content increases, DIF initially rises and then subsequently declines. When the fiber volume content is 0.10%, the DIF reaches its maximum value. This observation might be attributed to the impact of fiber content on crack development path and expansion degree. Based on this, when the rubber content is 20%, the optimal dosage of polypropylene fiber is 0.1%. Furthermore, under identical fiber content conditions, the DIF of SPRC specimens surpasses that of PRC. With the increase in fiber content, the DIF value of SPRC specimens increases by 3.20%, 1.47%, 2.14%, 2.21%, and 3.01% respectively compared to PRC, indicating that the integration of silica fume enhances the internal structure of concrete and improves the strain rate sensitivity of concrete materials.

In addition, under similar strain rates, the DIF^54^ of concrete with a high fiber content (4.55 kg/m^3^) is similar to the DIF of SPRC with 0.91 kg/m^3^ (0.1% dosage) of fibers in this study, which suggests that the addition of silica fume and rubber has a significant effect on enhancing the DIF.

## Microstructure analysis

### Effect of rubber on the microstructure of concrete

**Figure 19:**
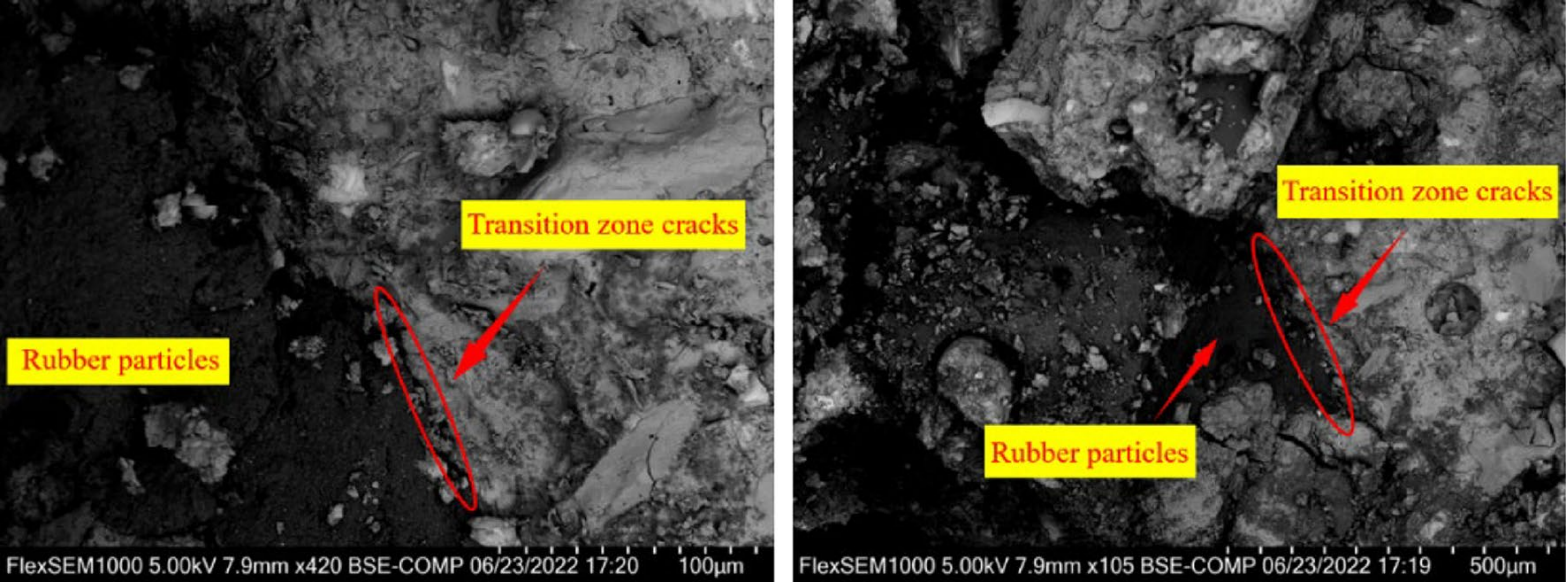
Aggregate interface of rubber concrete.

Figure 19 shows the SEM images of rubber concrete at a magnification of 1000 times. Through a detailed examination of PRC-1, it is evident that the cracks in the transition zone of rubber concrete are more pronounced. This is primarily due to the hydrophobic nature of rubber, which exhibits inferior water retention compared to sand. When rubber particles are incorporated into concrete, it results in the formation of numerous pores. Consequently, PRC-1 exhibits numerous cracks and experiences a corresponding reduction in strength. Additionally, the inclusion of rubber particles slows down the hydration reaction in concrete, leading to conspicuous cracks in the transition zone^55^. Because of rubber's poor water retention characteristics, PRC-1 experiences an increase in water loss during the hydration reaction, which limits the water requirement for the hydration process and results in a comparatively loose internal structure of the specimen during testing. The hydrophobicity of rubber also prevents hydration reactions from occurring on its surface. Consequently, stress is often concentrated near cracks close to the specimen, ultimately compromising the bearing capacity of concrete. When compared to normal concrete, PRC-1 exhibits reduced compressive strength, splitting tensile strength, and flexural strength.

### Effect of silica fume on the microstructure of concrete

**Figure 20:**
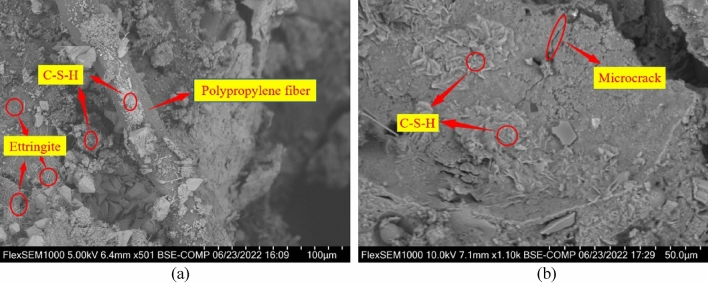
SEM image of aggregate interface with silica fume mixed with rubber concrete. (**a**) Specimens with 0% silica fume content, (**b**) Specimens with 10% silica fume content.

To analyze the microscopic morphology of concrete specimens with silica fume content of 0% and 10%, both were magnified by 1000 times. As displayed in Fig. 20a, there is a significant presence of needle-shaped ettringite crystals in the microstructure, with insufficient C–S–H gel filling between the crystals, leading to notable gaps. Conversely, Fig. 20b reveals an improved microstructure compactness in the specimen. It is noteworthy that there is no substantial accumulation of Ca(OH)_2_ crystals and the needle-shaped crystals are well-encapsulated by C–S–H gel. The primary reason for these observations lies in the surface effects and small size effects of silica fume. These properties enable it to participate in the hydration reaction of concrete, releasing heat and promoting further reactions^56^. Additionally, it effectively fills the pores between cement pastes, enhancing matrix compactness. Furthermore, silica fume exhibits strong pozzolanic activity, reacting with Ca(OH)_2_ produced by cement hydration to form C–S–H gel. This process not only repairs surface cracks in coarse aggregates but also enhances interfacial bonding strength, thereby improving mechanical properties^56^.

### Effect of polypropylene fiber on the microstructure of concrete

**Figure 21:**
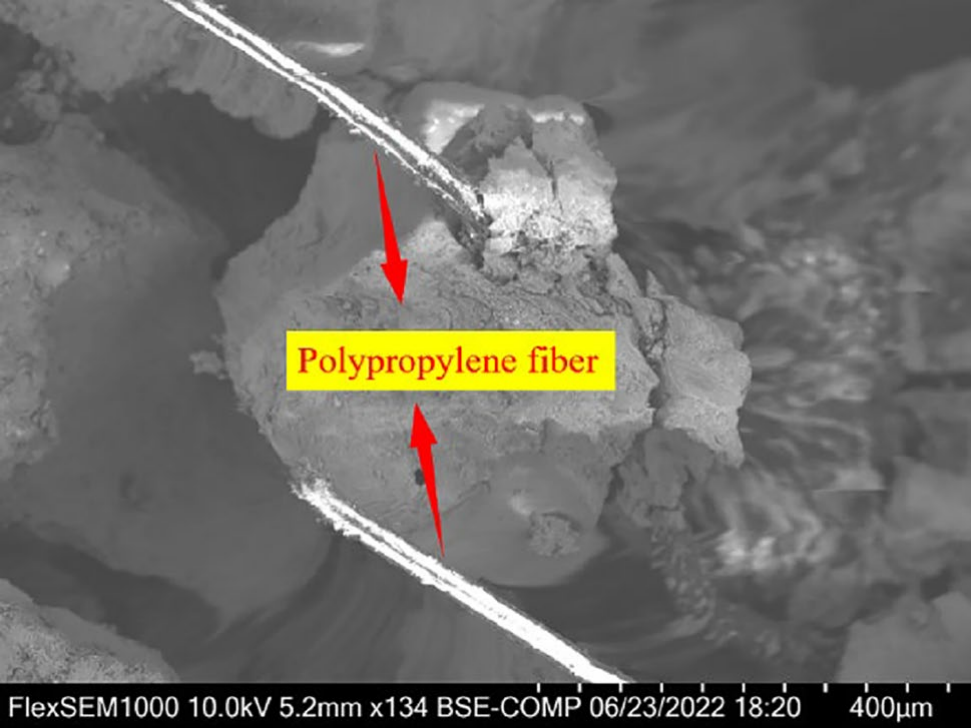
SEM photo of the bonding interface between polypropylene fiber and specimen matrix.

From the 1000 times magnification of the interface between the polypropylene fiber and the matrix in Fig. 21, it is evident that the fiber exhibits a flat appearance, uneven surface, and visible friction marks. These characteristics arise due to the fiber's involvement in tensile forces. Prior to concrete solidification, fibers are typically evenly distributed in a network within the concrete. This arrangement supports the settlement of coarse and fine aggregates, effectively mitigating surface cracks. Fibers act as barriers against concrete's post-hardening autogenous shrinkage, diminishing microcrack tensile deformation. Consequently, stress concentration at microcrack tips is reduced. When concrete sustains damage, fibers distributed at microcrack tips dissipate energy physically, preventing microcrack formation and propagation. This leads to an enhancement of concrete's internal structure. However, as fiber content increases, randomly distributed fibers within concrete bear a portion of the stress, leading to tensile deformation. This reduces concrete's autogenous shrinkage and transfers shrinkage-generated energy to individual fiber monofilaments. This transfer weakens the fibers' reinforcing effect. Similarly, large fiber dosages can cause 'fiber agglomeration' within the internal structure, increasing porosity and weakening the matrix's compactness and cohesiveness. This ultimately diminishes concrete's macroscopic strength^57^.

### Effect of silica fume-polypropylene fiber interaction on the microstructure of concrete

**Figure 22:**
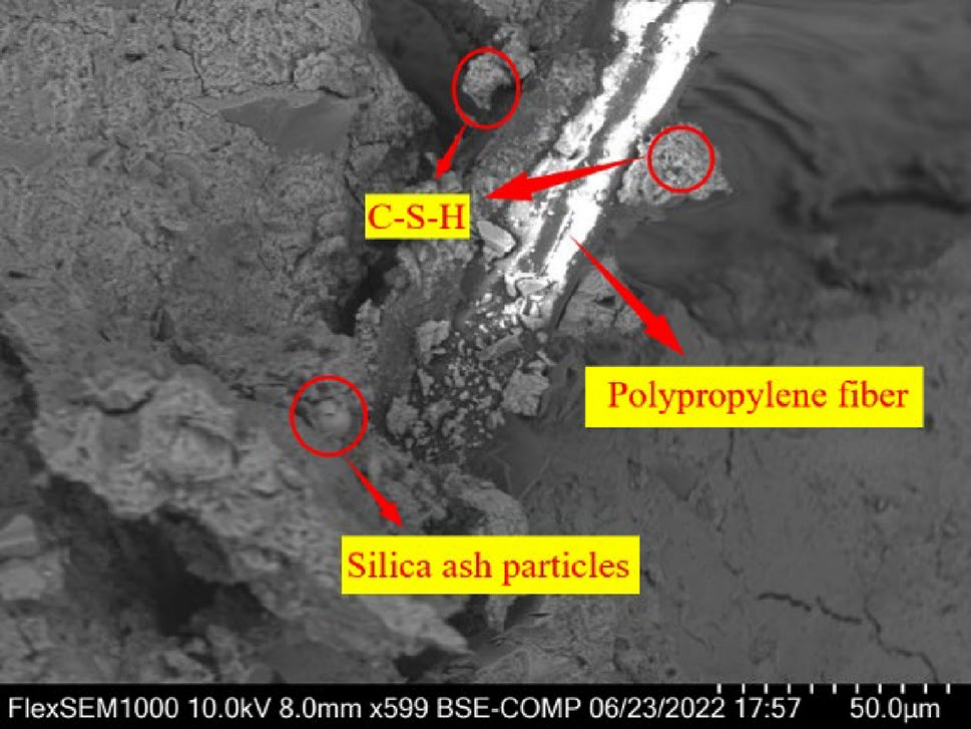
SEM photo of the bonding interface between polypropylene fiber and specimen matrix.

The microstructure of rubber concrete specimens modified with silica fume and polypropylene fibers is vividly illustrated in Fig. 22. This figure reveals that silica fume, owing to its smaller particle size, effectively fills smaller pores. Consequently, it enhances the adhesion between the polypropylene fiber and the rubber concrete matrix, resulting in improved compactness of the rubber concrete. Additionally, silica fume, characterized by its high specific surface area, reacts swiftly with Ca(OH)_2_ to generate hydration heat. This heat facilitates the hydration of cement, leading to an increased production of hydration products, thereby augmenting the concrete's strength. When subjected to a load, the specimen's supportive skeleton structure composed of randomly distributed fibers within the concrete, collaborates with the silica fume. This combination effectively hinders the formation and propagation of cracks, thereby enhancing the mechanical properties of the concrete^58^. Hence, an optimal blend of silica fume and polypropylene fibers can significantly mitigate the occurrence and progression of cracks in rubber concrete. This also elevates the mechanical properties of rubber concrete, providing a robust foundation for the production of high-strength, high-toughness rubber concrete.

## Conclusion

1. Both normal concrete and rubber concrete exhibited significant aggregate fracturing after failure, and the specimens were unable to maintain integrity. Upon the addition of polypropylene fibers, although numerous cracks appear on the specimen surface, only a few fragments detach, demonstrating more ductile failure characteristics.
2. As the polypropylene fiber content increased, the compressive strength, splitting tensile strength, and flexural strength of the specimens initially increased and then decreased. At a fiber content of 0.1%, all three strength parameters reached their maximum values, offering an increase of 11%, 20.8%, and 19.3% respectively compared to rubber concrete without polypropylene fiber modification. Furthermore, the incorporation of 10% silica fume to concrete with a polypropylene fiber content of 0.1% significantly enhanced the mechanical properties of the specimens, leading to improvements of 13.7%, 26.6%, and 16.6% in the three strength parameters compared to the specimens without silica fume.
3. In dynamic compression tests, with constant silica fume content, the dynamic compressive strength of the specimens initially increased and then decreased as the fiber content increased. Notably, when the silica fume and polypropylene fiber contents of the rubber concrete were 10% and 0.1% respectively, the dynamic compressive strength of the silica fume-polypropylene rubber concrete reached its maximum value of 58.61 MPa.
4. SEM analysis revealed that polypropylene fibers randomly distributed three-dimensionally to form a skeleton structure within the concrete. Meanwhile, the silica fume filled the pores between the cement paste, reducing matrix porosity. Additionally, it reacts with Ca(OH)_2_ produced during cement hydration to form C–S–H gel, further repairing cracks on the surface of coarse aggregates. These synergistic effects contribute to a more dense concrete structure and greater adhesion.

## Data Availability

The datasets used and/or analyzed during the current study available from the corresponding author on reasonable request.
